# Wen-Luo-Tong Decoction Attenuates Paclitaxel-Induced Peripheral Neuropathy by Regulating Linoleic Acid and Glycerophospholipid Metabolism Pathways

**DOI:** 10.3389/fphar.2018.00956

**Published:** 2018-08-28

**Authors:** Fei-ze Wu, Wen-juan Xu, Bo Deng, Si-da Liu, Chao Deng, Meng-yu Wu, Yu Gao, Li-qun Jia

**Affiliations:** ^1^Graduate School, Beijing University of Chinese Medicine, Beijing, China; ^2^Research Center for Chinese Medical Analysis and Transformation, Beijing University of Chinese Medicine, Beijing, China; ^3^Department of Traditional Chinese Medicine Oncology, China-Japan Friendship Hospital, Beijing, China

**Keywords:** paclitaxel, peripheral neuropathy, metabolomics, Wen-Luo-Tong (WLT), TCM, UHPLC-MS/MS

## Abstract

Chemotherapy-induced peripheral neuropathy (CIPN) is a serious dose-limiting toxicity of many anti-neoplastic agents, especially paclitaxel, and oxaliplatin. Up to 62% of patients receiving paclitaxel regimens turn out to develop CIPN. Unfortunately, there are so few agents proved effective for prevention or management of CIPN. The reason for the current situation is that the mechanisms of CIPN are still not explicit. Traditional Chinese Medicine (TCM) has unique advantages for dealing with complex diseases. Wen-Luo-Tong (WLT) is a TCM ointment for topical application. It has been applied for prevention and management of CIPN clinically for more than 10 years. Previous animal experiments and clinical studies had manifested the availability of WLT. However, due to the unclear mechanisms of WLT, further transformation has been restricted. To investigate the therapeutic mechanisms of WLT, a metabolomic method on the basis of UPLC- MS was developed in this study. Multivariate analysis techniques, such as principal component analysis (PCA) and partial least squares discriminate analysis (PLS-DA), were applied to observe the disturbance in the metabolic state of the paclitaxel-induced peripheral neuropathy (PIPN) rat model, as well as the recovering tendency of WLT treatment. A total of 19 significant variations associated with PIPN were identified as biomarkers. Results of pathway analysis indicated that the metabolic disturbance of pathways of linoleic acid (LA) metabolism and glycerophospholipid metabolism. WLT attenuated mechanical allodynia and rebalanced the metabolic disturbances of PIPN by primarily regulating LA and glycerophospholipid metabolism pathway. Further molecular docking analysis showed some ingredients of WLT, such as hydroxysafflor yellow A (HSYA), icariin, epimedin B and 4-dihydroxybenzoic acid (DHBA), had high affinity to plenty of proteins within these two pathways.

## Introduction

Chemotherapy-induced peripheral neuropathy (CIPN) is a common treatment-related adverse effect of many anti-neoplastic agents. It is a serious dose-limiting toxicity characterized by distal, symmetrical, sensory peripheral neuropathy. These sensory neuropathy symptoms generally present as paraesthesia, numbness and/or pain, which severely affect the patient's quality of life (QOL). Although recent advances in cancer treatment have led to prolonged survival of patients, many treatment-related adverse effects remain unsolved (Wolf et al., 2008; Hershman et al., 2011, 2014). There are few established agents recommended for the prevention or management of CIPN to cancer survivals undergoing anti-neoplastic treatment with neurotoxic agents (Hershman et al., 2014), such as paclitaxel, oxaliplatin, vinorelbine, et al. Paclitaxel, as an effective anti-neoplastic agent, is widely used as first-line treatment for various types of cancer, including of breast, ovarian, and non-small cell lung cancer. However, CIPN is a significant problem for patients receiving paclitaxel regimens. Up to 62% of patients turn out to develop CIPN after paclitaxel regimens (Lee and Swain, 2006; Argyriou et al., 2008; Reyes-Gibby et al., 2009). It has been reported that mitochondrial damage (André et al., 2000; Bernardi et al., 2006; Flatters and Bennett, 2006; Martin et al., 2009), intraepidermal nerve fibers degeneration (Flatters and Bennett, 2006), ion channels alteration (Xiao et al., 2007; Nieto et al., 2008; Zhang et al., 2014), transient receptors potential (TRP) family (Alessandri-Haber et al., 2008; Materazzi et al., 2012), inflammation and immune status (Ledeboer et al., 2007; Dutra et al., 2015; Li D. et al., 2015) are associated with paclitaxel-induced peripheral neuropathy (PIPN). However, the mechanisms remain unclear. An explicit mechanism is the precondition of discovery for eutherapeutic drug or treatment, thus it's very important and urgent to understand its mechanism.

After more than several decades of efforts, the treatment against cancer and its complications still appears far from being satisfying. It has gradually become clear that the “one gene-one target-one drug” model has failed for drug discovery. It appears that monotherapy strategy will be replaced by rational combination targeted therapy (Haefner, 2006). It is necessary to analyze the systems' response to drug treatments, not just one target or pathway. Therefore, is very important to reveal the abnormal metabolisms through a systematic perspective for understanding the mechanisms of disease, as well as for developing drugs. Metabolomics is a collection of powerful tools for detecting, identifying and quantifying the endogenous metabolites that are involved in the metabolisms, and then interpreting biological changes of the internal environment (Nicholson and Lindon, 2008). It has been increasingly used in the researches aiming to explore the mechanisms of diseases or treatments, as it could provide comprehensive metabolic information of organisms (Weckwerth, 2003, 2010; Glinski and Weckwerth, 2006; Fang and Gonzalez, 2014; Wang et al., 2015).

Currently, drug combinations and multi-target therapies have been proposed as a promising therapeutic strategy for improving anti-neoplastic effects, and relieving the side effects. Traditional Chinese Medicine (TCM) deals with diseases from the perspective of holism. The TCM decoctions are commonly composed of several herbs with complex constituents of compound. Metabolomics has been used as important technique for interpreting pharmacological mechanisms of TCM formulae under the guidance of systematic theory (Li et al., 2011; Li and Zhang, 2013). Indeed, there have been an increasing number of TCM studies applying metaboliomics (Lu et al., 2014; Cao et al., 2015; Wang et al., 2015, 2017). Wen-Luo-Tong (WLT) is an herbal formula ointment for topical application. It had been applied to prevention and management for CIPN in clinical practice for more than 10 years. The formula was constituted by epimedium herb, geranium wilfordii, cassia twig, and carthamus tinctorius (Table 1). The components are supposed to act synergistically to achieve the TCM treatment principle of Warming and Activating Meridian, Promoting Blood Circulation and Alleviating Pain. Moreover, previous clinical trial and animal experiment manifested it had effect of analgesic and neuroprotection over CIPN. The clinical trial showed that after 7 days WLT treatment, numerical rating scale (NRS) in trial group was significantly decreased while control group did not. And the response rate of pain relief was 85.07% in trial group while 44.12% in control group (*p* < 0.01). In addition, symptoms of 75.00% subjects in trial group were improved by grading, while 35.29% in control group. The animal experiment showed WLT treatment alleviated oxaliplatin-induced mechanical allodynia and mechanical hyperalgesia. Degenerations in the nuclear, and nucleolar areas of neurons in dorsal root ganglion (DRG) were attenuated. In the spinal dorsal horn, hypertrophy and activation of glial fibrillary acidic portein (GFAP) positive astrocytes were averted, and the level of GFAP mRNA decreased significantly (Lou et al., 2008, 2014; Deng et al., 2017). Therefore, decipher the mechanisms of WLT based on metabolomics will provide a global insight into the actions of neurotoxic agents and WLT, which helps to understand the mechanisms and further helps to discovery new drug or treatment.

**Table 1:** Constituents of WLT.

**Botanical name**	**English name**	**Used plant parts**	**Weight per dose (g)**
*Epimedium brevicornum Maxim*.	Epimedium herb	Dried overground parts	30
*Geranium wilfordii Maxim*.	Geranium wilfordii	Dried overground parts	20
*Cinnamomum cassia Presl*.	Cassia Twig	Dried twig	15
*Carthamus tinctorius L*.	Carthamus tinctorius	Dried flower	10

Our study aimed to investigate the metabolic disturbances of PIPN through untargeted metabolomic assays. Additionally, taking WLT as probe, metabolomic alterations were analyzed to provide some clues for CIPN mechanism explorations.

## Materials and methods

### Paclitaxel and WLT preparation

Paclitaxel (6 mg/ml, Beijing Union Pharmaceuticals, Beijing, China) was diluted with saline (1:3) (Li D. et al., 2015). WLT decoction was authenticated by both Ethics Committee and Pharmaceutical Department of China-Japan Friendship Hospital. Herbs of WLT (Table 1) were purchased from pharmacy of China-Japan Friendship Hospital (Beijing, China). WLT decoction was prepared into granula with individual package (composes and dosage of each package showed in Table 1) by the Pharmaceutical Department of China-Japan Friendship Hospital, following the technological process shown in Supplementary Figure 1. (Manufacturer production lot number: 201610211416).

### Animal experiments and sample collection

Female Sprague-Dawley rats weighing 160–200 g were purchased from Beijing Vital River Laboratory Animal Technology Co., Ltd. (Beijing, China). All rats were housed in automatically controlled environmental conditions, using a 12 h light–dark cycle (lights on from 08:00 to 20:00) with free access to food and water. All animal experiment protocols were approved by the Animal Care and Welfare Committee of China-Japan Friendship Hospital (Beijing, China), approval No. 160105. The investigation was conducted in accordance with the ethical principles of animal use and care.

After 3 days adaption, rats were randomly allocated into three groups as follows: PTX, WLT, Control. Animals of PTX and WLT were intraperitoneally (i.p.) injected (8 mg/kg, cumulative dose of 24 mg/kg) on 3 alternate days (d1, 4, 7) (Li D. et al., 2015). Animals of Control received an equivalent volume of saline. One package of WLT (Table 1) was dissolved into 1,000 ml deionized water (40°C) before application. Animals in WLT group shared one batch of WLT solution together. Animals took pediluvium of WLT or water for 30 min, twice a day (Supplementary Figure 4). The intervention was initiated 1 day before paclitaxel administration and lasted for 11 days (d0-10). Rats in PTX and Control took pediluvium of deionized water. The route of operation was as follow: behavioral test, pediluvium, intraperitoneal injection (Deng et al., 2016).

Mechanical paw withdrawal threshold (PWT) was tested before, during, and after paclitaxel treatment (d0, 2, 4, 6, 8, and 10) by an experimenter blinded to treatment groups (Li D. et al., 2015). Rats were placed in elevated plexiglas chambers upon a wire mesh floor and allowed to acclimate for 15 min prior to measuring the PWT. PWT was tested using calibrated von Frey filaments according to the “up and down” method (Dixon, 1980; Chaplan et al., 1994). The development of mechanical allodynia was evidenced by a significant (p < 0.05) reduction in mean absolute PWT (g) at forces that failed to elicit withdrawal responses before treatment (baseline or D0). PWT testing was done prior to any other administration or intervention.

**Figure 1:**
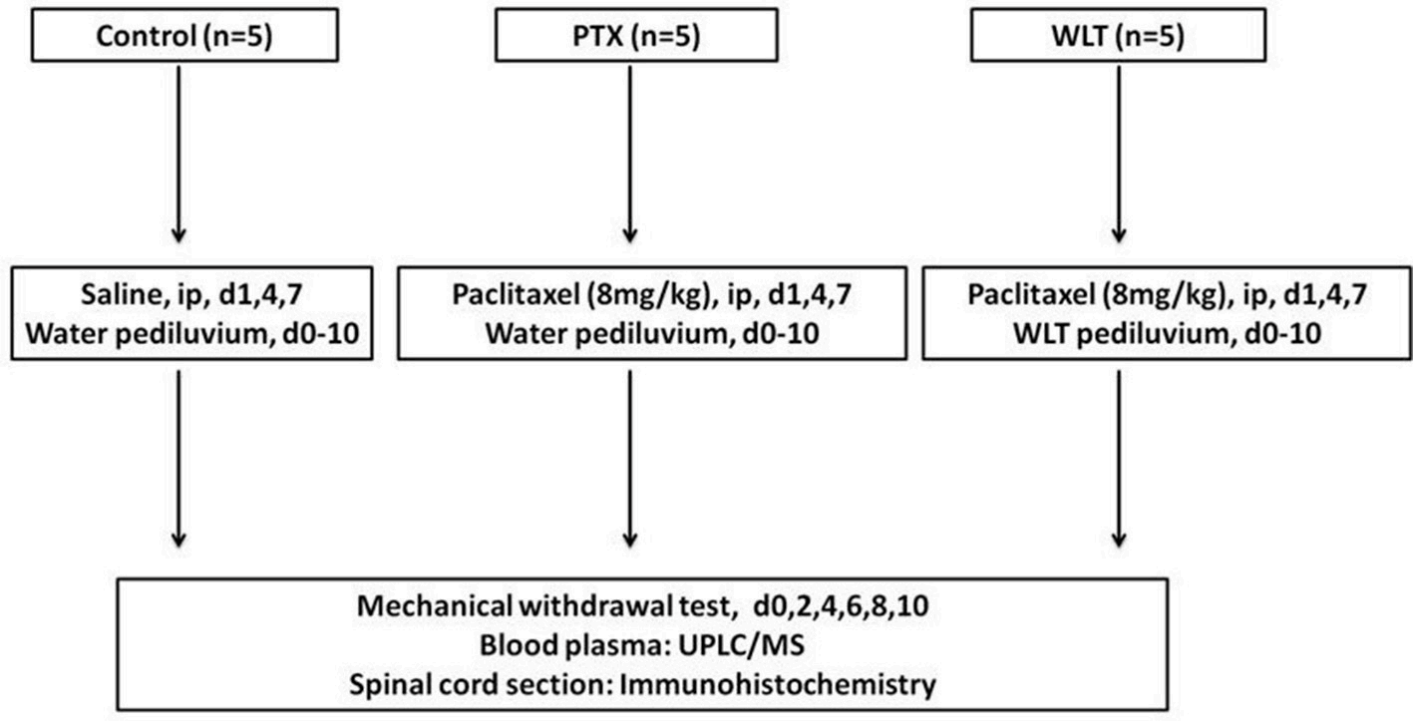
Experimental design. Animals of Control, PTX and WLT received paclitaxel/saline injection and WLT/water pediluvium during the experiment. The PIPN model was constructed by administration of paclitaxel at 8 mg/kg by intraperitoneal injection on d1, 4, 7. WLT solution was given tropically by pediiluvium twice a day for 11 consecutive days (d0-10). The behavior assay was monitored every other day (d0, 2, 4, 6, 8, 10). The rats were finally euthanized on day 10. Plasma and spinal cord samples were collected for preparation and analysis.

Blood and tissue samples were collected on day 10 after tests and interventions. Rats were anesthetized with intraperitoneal injection of sodium pentobarbital (50 mg/kg). Blood samples were centrifuged at 3,000 rpm for 10 min. Then 500 μL plasma was extracted. All the samples were stored in a −80°C freezer for further analysis. L4–6 spinal cord segments were removed, fixed in 4% paraformaldehyde overnight, embedded in paraffin and cut into 5 μm thickness sections. The scheme of the whole experiment was shown in Figure 1.

### Immunohistochemistry

Sections of the L4–6 spinal cord were processed for immunohistochemistry using a primary antibody against Chemokine (C-X3-C motif) ligand 1 (CX3CL1) (1:200, Bosterbio, China). Following incubation overnight at 4°C, sections were incubated in a horseradish peroxidase-conjugated secondary antibody for 4 h at room temperature. The color was developed with 3,3′-diaminobenzidine (DAB).

### Instrumentation for LC-MS

An ultra-performance liquid chromatography-electrospray ionization-mass spectrometer (UPLC-ESI-MS) was used. It was equipped with hybrid quadrupole Orbitrap mass spectrometer and ThermoFisher Scientific Q Exactive (Thermo Fisher Scientific, Inc., Waltham, MA, United States). The sample solutions were separated using a HSS C_18_ column (2.1 × 100 mm, 1.7 μm, Waters) at 45°C. For C_18_ separation, mobile phase A was water and mobile phase B was acetonitrile. Both A and B contained 0.1% formic acid and 1 mmol/L ammonium acetate. The flow rate was 300 μL/min and the injection volume was 1 μL. The positive and negative HESI-II spray voltages were 3.7 and 3.5 kV, respectively, the heated capillary temperature was 320°C, the sheath gas pressure was 30 psi, the auxiliary gas setting was 10 psi, and the heated vaporizer temperature was 300°C. Both the sheath gas and the auxiliary gas were nitrogen. The collision gas was also nitrogen at a pressure of 1.5 mTorr. The parameters of the full mass scan were as follows: a resolution of 70,000, an auto gain control target under 1 × 10^6^, a maximum isolation time of 50 ms, and an m/z range 150–1,500. The calibration was customized for the analysis of Q Exactive to keep the mass tolerance of 5 ppm. The parameters of the dd-MS2 scan were as follows: a resolution of 17,500, an auto gain control target under 1 × 10^5^, a maximum isolation time of 50 ms, a loop count of top 10 peaks, an isolation window of m/z 2, normalized collision energy of 30 V and an intensity threshold under 1 × 10^5^. The LC-MS system was controlled using Xcalibur 2.2 SP1.48 software (Thermo Fisher Scientific, United States), and data were collected and processed with the same software.

All data obtained from the assays in the two systems of both positive and negative ion modes were processed using Progenesis QI data analysis software (Nonlinear Dynamics, Newcastle, UK) for imputing raw data, peak alignment, picking, and normalization to produce peak intensities for retention time (tR) and m/z data pairs. The ranges of automatic peak picking for the C_18_ assays were between 0.7 and 19 min. Then, the adduct ions of each “feature” (m/z, tR) were deconvoluted, and these features were identified in the human metabolome database (HMDB, http://www.hmdb.ca/) and Lipidmaps (http://www.lipidmaps.org/).

### Quality control

To monitor the system's stability and performance as well as the reproducibility of the samples, quality control samples (QCs) were prepared by pooling equal volumes of each sample. The pretreatment of QCs was in accord with real samples. For repeatable metabolic analyses, three features of the analytical system must be stable: (i) retention time, (ii) signal intensity and (iii) mass accuracy. In this study, three QCs were continuously injected at the beginning of the run. QCs are then injected at regular intervals throughout the analytical run in order to provide data from which repeatability can be assessed. The features were selected based on their coefficients of variation (CVs) with QCs; features with CVs over 15 % were eliminated.

### Statistical analysis

Statistical analysis of behavioral test was performed using SPSS 19.0 (IBM, USA). Results of pathological scores were analyzed using IPP 6.0 (Intel, USA). The multivariate data analysis, principal component analysis (PCA) and partial least squares discriminate analysis (PLS-DA) were conducted by EZinfo 3.0 (Umetrics, Sweden). Metabolism pathway analysis was conducted by MetaboAnalyst 3.0 (http://www.metaboanalyst.ca/). Enzymes related to metabolites were collected by HMDB 4.0 (http://www.hmdb.ca/). Protein-protein interactions with confidence >0.9 in the STRING 10.5 (https://string-db.org) were used to construct a network containing relationships between enzymes and proteins. The structures of WLT ingredients were downloaded from PubChem (http://pubchem.ncbi.nlm.nih.gov). The structures of proteins were obtained from the Protein Data Bank (PDB, https://www.rcsb.org/). Protein structures not available from PDB was homology modeled by (https://www.swissmodel.expasy.org) docking was carried out with Discovery Studio 3.5 (BIOVIA, USA). Parametric data was analyzed by ANOVA or Student's *t*-test for, and non-parametric data by Mann–Whitney *U*-test or Kruskal-Wallis. Differences were considered the results with *P*-values < 0.05.

## Results

### WLT attenuated paclitaxel-induced peripheral neuropathy

**Figure 2:**
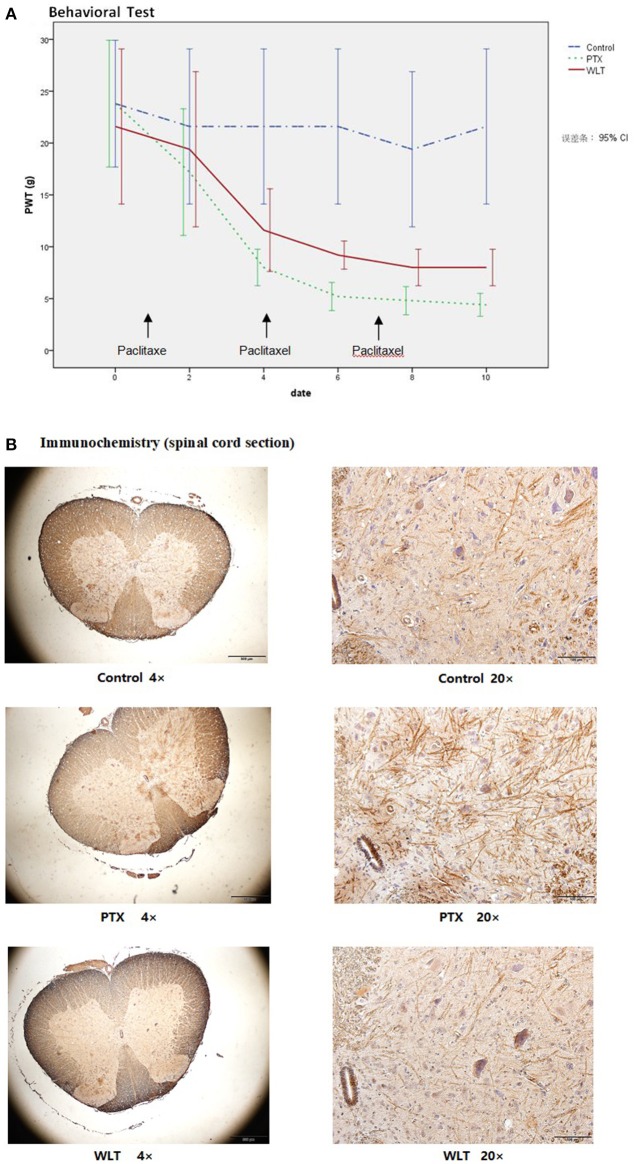
WLT attenuated Paclitaxel-induced Peripheral Neuropathy. **(A)** Paw withdraw threshold (PWT) of PTX and WLT group was decreased significantly after injection of paclitaxel (8 mg/kg, i.p.). Statistical difference had been shown since day 4. Compared with PTX group, WLT attenuated the mechanical allodynia. Significant difference was shown since day 6 (*p* < 0.05). **(B)** Immunochemistry staining showed that paclitaxel induced up-regulation of CX3CL1 expression in neurons and dendrites. The expression of CX3CL1 in WLT was down-regulated.

Consistent with previous studies (Huang et al., 2014; Li D. et al., 2015), administration with paclitaxel (3 × 8 mg/kg, cumulative dose 24 mg/kg, equivalent to average dose of clinical practice) induced marked mechanical allodynia (Figure 2A). The PWT of both WLT and PTX was greatly decreased compared with Control group. However, co-administrated with WLT alleviated PWT decrease. PWT of PTX group was decreased significantly from 20.5 ± 5.88 to 4.5 ± 0.93 g. Statistical difference between PTX and Control had been shown since day 4. While that of WLT group was from 20.5 ± 5.88 to 8.0 ± 1.07 g. Statistical difference between PTX and WLT had been shown since day 6. According to previous studies, it had been revealed that up-regulation of CX3CL1 was involved with the neuropathic pain induced by nerve injury. Immunochemistry staining of our study showed that paclitaxel increased the expression of CX3CL1 in neurons and dendrites of spinal cord sections. Meanwhile, compared with PTX group, CX3CL1 expression in WLT was markedly down-regulated (Figure 2B). The results of behavioral test and immunochemistry demonstrated that WLT attenuated paclitaxel-induced peripheral neuropathy.

### Metabolic disturbances of paclitaxel-induced peripherial neuropathy

PCA manifested paclitaxel induced obvious metabolic disturbances. QCs were applied in assessing system stability. The PCA plots showed a clear discrimination between Control and PTX groups, while QCs highly clustered (Supplementary Figure 2), indicating that the PIPN model was successfully duplicated and our analytical method was of good reproducibility and stability.

**Figure 3:**
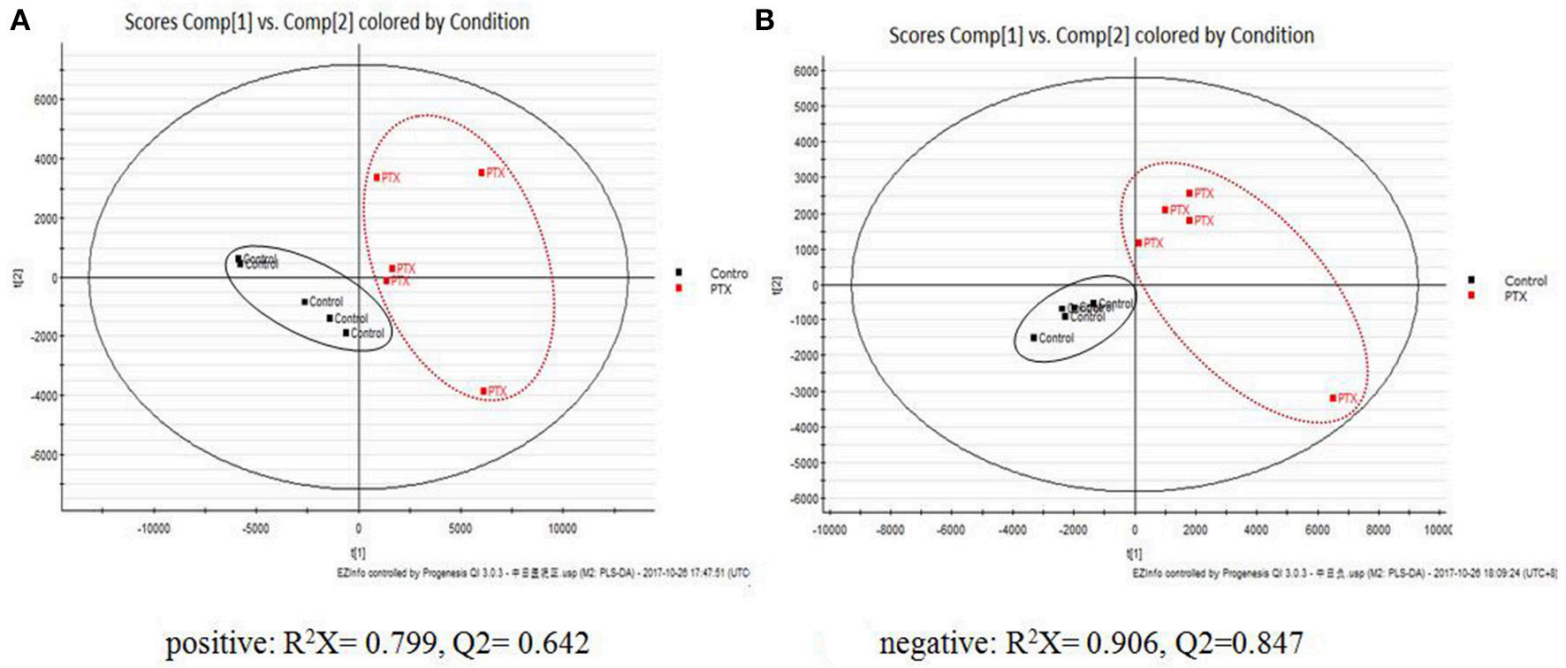
PLS-DA Plots of PIPN Metabolic Disturbances. The PLS-DA plots showed clear separation between Control and PTX. **(A)** Positive: *R*^2^*X* = 0.799, *Q*^2^ = 0.642. **(B)** Negative: *R*^2^*X* = 0.906, *Q*^2^ = 0.847.

**Figure 4:**
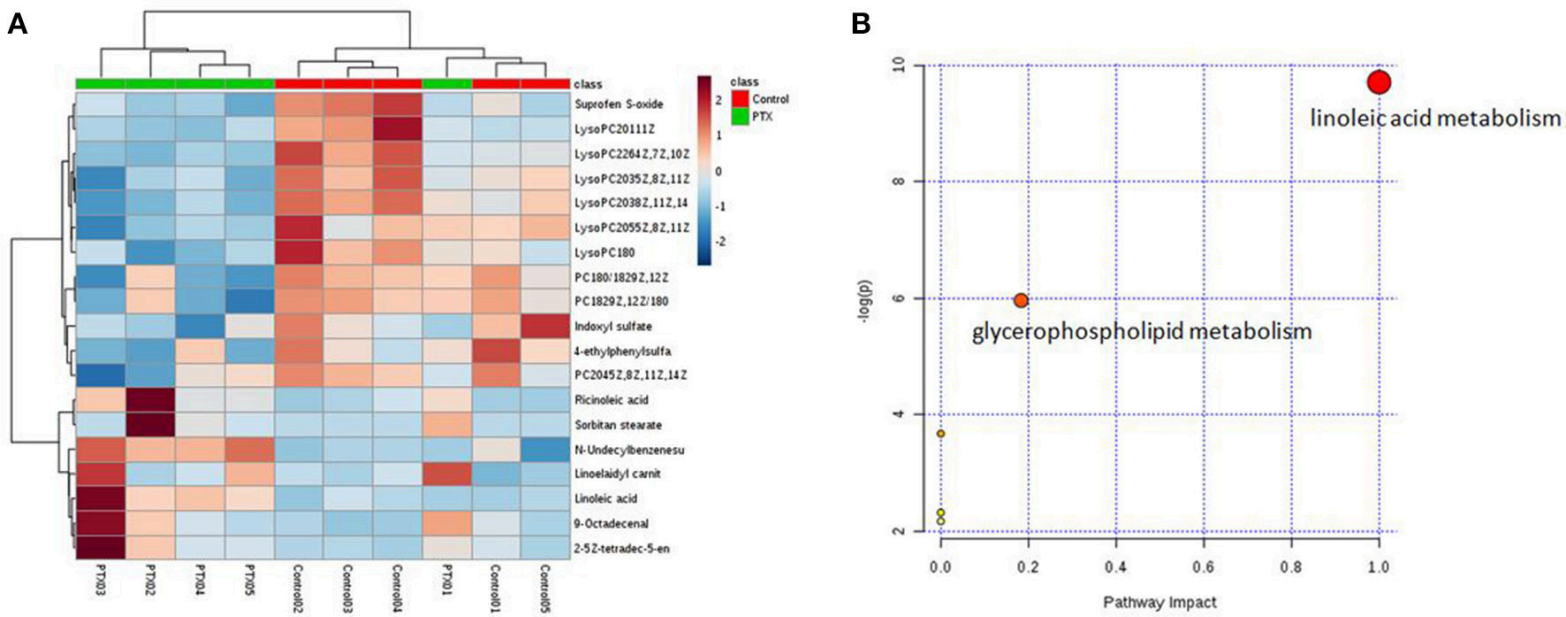
Metabolites variation analysis. **(A)** Heatmap of metabolite concentrations. Heatmap showed the relative concentrations of differential metabolites in each sample. **(B)** Metabolism Pathway Affected by Paclitaxel Treatment. The result of the significant metabolites analysis showed that paclitaxel primarily disturbed the linoleic acid and glycerophospholipid metabolisms. Linoleic acid metabolism was a preferred target pathway of paclitaxel (impact 1.0).

To identify the significant metabolic disturbances of PIPN, PLS-DA models of PTX and Control were further performed. The score plots demonstrated a satisfactory separation between Control and PTX (Figure 3A, positive: *R*^2^*X* = 0.799, *Q*^2^ = 0.642; Figure 3B, negative: *R*^2^*X* = 0.906, *Q*^2^ = 0.847). The variable importance in the projection (VIP) value of each variable in the model was ranked according to its contribution to the classification. The VIP list of the retention time-exact mass pairs was obtained from the PLS-DA using EZinfo 3.0. To select the potential biomarkers worthy of preferential study in the next step, these differential metabolites were validated using the Student's *t*-test. The critical *p*-value was set to 0.05 for the significantly different variables in this study. Following the criteria above, 19 differential metabolites were identified (Table 2). Most of the metabolites were lipids (3 phosphatidylcholines and 6 lysophospholipid), fatty acid or organic acid (LA, N-Undecylbenzenesulfonic acid, and Ricinoleic acid), ketones ((±)-(Z)-2-(5-Tetradecenyl) cyclobutanone and Suprofen S-oxide). To show the variation trend of the significant metabolites, a heatmap was drawn according to relative concentrations of the differential metabolites (Figure 4A). As shown in Figure 4A, 12 metabolites were down-regulated in PTX, including all the PCs and lysoPCs, Indoxyl sulfate, Suprofen S-oxide, and 4-ethylphenylsulfate. The other 7 metabolites were up-regulated. These results revealed the potential role of lipids in the metabolic response to paclitaxel.

**Table 2:** Significantly differential metabolites.

	**HMDB Code**	**name**	**m/z**	**Retention time (min)**	***p*-value**	**VIP**	**FC**
(+)	HMDB06461	Linoelaidyl carnitine	424.342	7.49	0.04	1.04	2.093
	HMDB10391	LysoPC(20:1(11Z))	550.397	9.32	0.02	3.13	2.323
	HMDB10393	LysoPC(20:3(5Z,8Z,11Z))	546.356	8.25	0.02	2.63	1.983
	HMDB10384	LysoPC(18:0)	546.353	9.07	0.02	1.02	1.743
	HMDB10394	LysoPC(20:3(8Z,11Z,14Z))	546.355	8.18	0.05	3.94	1.763
	HMDB10404	LysoPC(22:6(4Z,7Z,10Z,13Z,16Z,19Z))	568.338	8.26	0.001	1.14	3.421
	HMDB29888	Sorbitan stearate	448.363	8.59	2.06E-11	1.17	↑↑
	HMDB30965	9-Octadecenal	284.295	10.45	0.04	1.10	2.345
	HMDB37543	(±)-(Z)-2-(5-Tetradecenyl) cyclobutanone	282.279	9.75	0.04	2.53	4.452
(–)	HMDB00673	Linoleic acid	279.233	9.73	0.05	1.38	2.602
	HMDB00682	Indoxyl sulfate	212.002	4.78	0.03	3.02	2.010
	HMDB08039	PC(18:0/18:2(9Z,12Z))	830.593	7.86	0.01	2.58	1.175
	HMDB08135	PC(18:2(9Z,12Z)/18:0)	830.592	8.12	0.01	1.66	1.155
	HMDB08431	PC(20:4(5Z,8Z,11Z,14Z)/18:0)	854.592	8.52	0.05	4.27	1.235
	HMDB10397	LysoPC(20:5(5Z,8Z,11Z,14Z,17Z))	586.316	7.58	0.05	1.71	1.533
	HMDB32549	N-Undecylbenzenesulfonic acid	311.169	10.32	0.02	1.04	1.122
	HMDB34297	Ricinoleic acid	297.243	8.50	0.001	1.76	3.391
	HMDB60924	Suprofen S-oxide	321.044	5.64	0.05	1.04	2.390
	HMDB62551	4-ethylphenylsulfate	201.022	5.66	0.05	2.49	1.970

To further investigate the metabolism pathway affected by paclitaxel treatment, the significant metabolites were analyzed using Metaboanalyst 3.0 (http://www.metaboanalyst.ca/), a free, web-based tool that combines metabolites with the pathway analysis to help researchers to identify the relevant pathways under their study conditions (Xia et al., 2015; Xia and Wishart, 2016). Metabolites in Table 2 were input into Metaboanalyst and the metabolic networks were depicted (Figure 4B). The result showed that paclitaxel primarily disturbed the LA metabolism (impact 1.0) and glycerophospholipid metabolism (impact 0.18), which was in accord with published literatures (Patwardhan et al., 2010; Wang et al., 2013; Sisignano et al., 2016). And LA metabolism was considered as a preferred target pathway of paclitaxel due to its significant impact score. LA, PC(20:4(5Z,8Z,11Z,14Z)/18:0), PC(18:0/18:2(9Z,12Z)), PC(18:2(9Z,12Z)/18:0) were key metabolites of LA metabolism pathway. While LysoPC(20:5(5Z,8Z,11Z, 14Z,17Z)), LysoPC(22:6(4Z,7Z,10Z,13Z,16Z,19Z)), LysoPC(20:3 (8Z,11Z, 14Z)), LysoPC(18:0), LysoPC(20:3(5Z,8Z,11Z)), LysoPC(20:1(11Z)) were key metabolites of glycerophospholipid metabolism pathway (Supplementary Figure 3). Thus, paclitaxel induced peripheral neuropathy by primarily targeting LA metabolism and glycerophospholipid metabolism pathways.

### WLT rebalanced the metabolic disturbances

**Figure 5:**
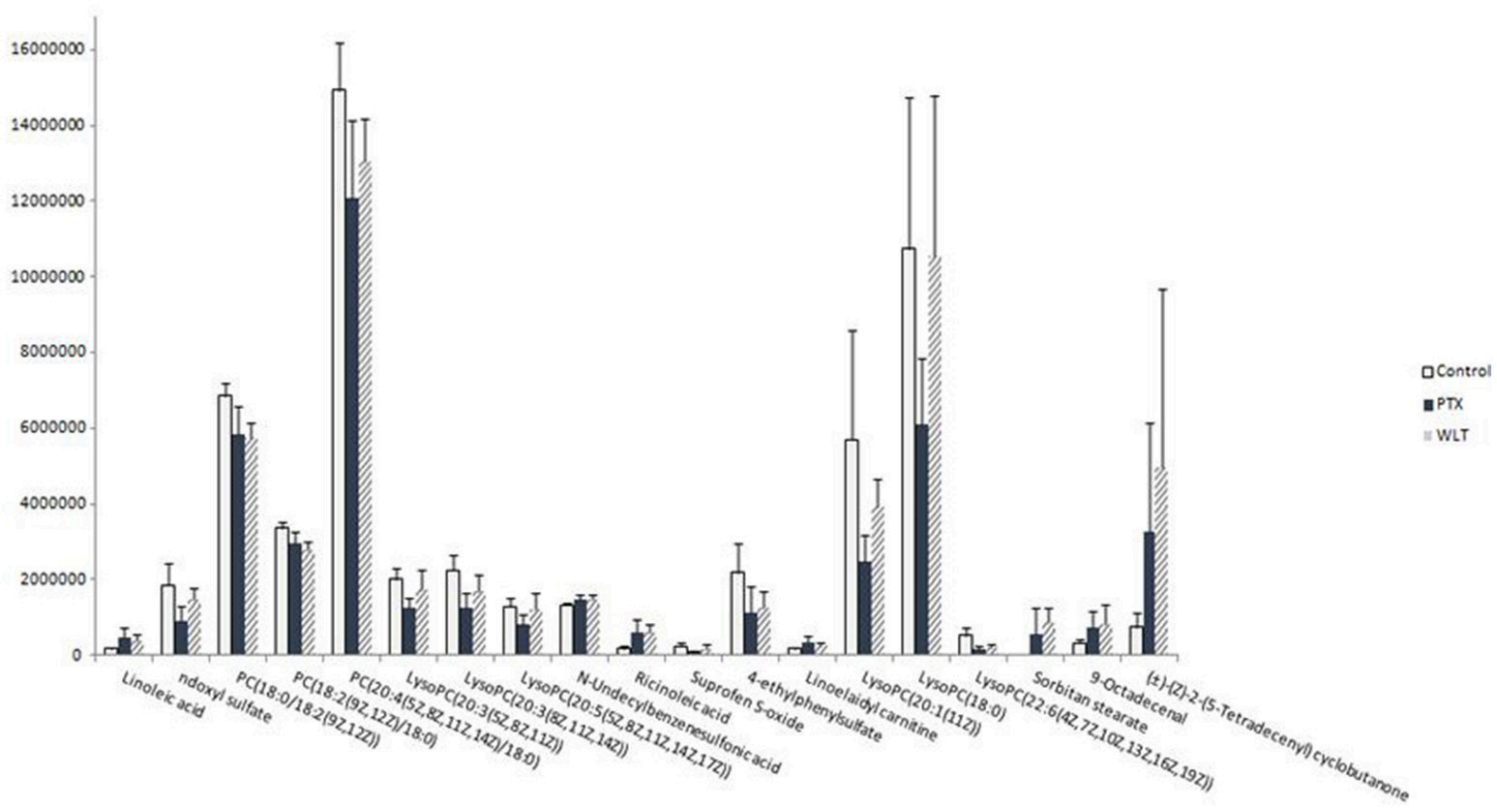
Effects of WLT on Regulating the Metabolic Disturbances. WLT rebalanced most metabolic disturbances caused by paclitaxel.

WLT treatment rebalanced the alteration of most metabolites disturbed by paclitaxel. The expression of 14 disturbed metabolites appeared to be rebalanced, including most of the key metabolites in LA metabolism and glycerophospholipid metabolism pathways (Figure 5). Especially, LA, PC(20:4(5Z,8Z,11Z,14Z)/18:0), LysoPC(20:3(5Z,8Z,11Z)), LysoPC(20:3(8Z,11Z,14Z)), LysoPC(20:5(5Z,8Z,11Z,14Z,17Z)), LysoPC(20:1(11Z)), LysoPC(22:6(4Z,7Z,10Z,13Z,16Z,19Z)), LysoPC(18:0) were rebalanced after WLT treatment. In addition, indoxyl sulfate, linoelaidyl carnitine, 4-ethylphenylsulfate, and Suprofen S-oxide were also regulated by WLT treatment.

**Figure 6:**
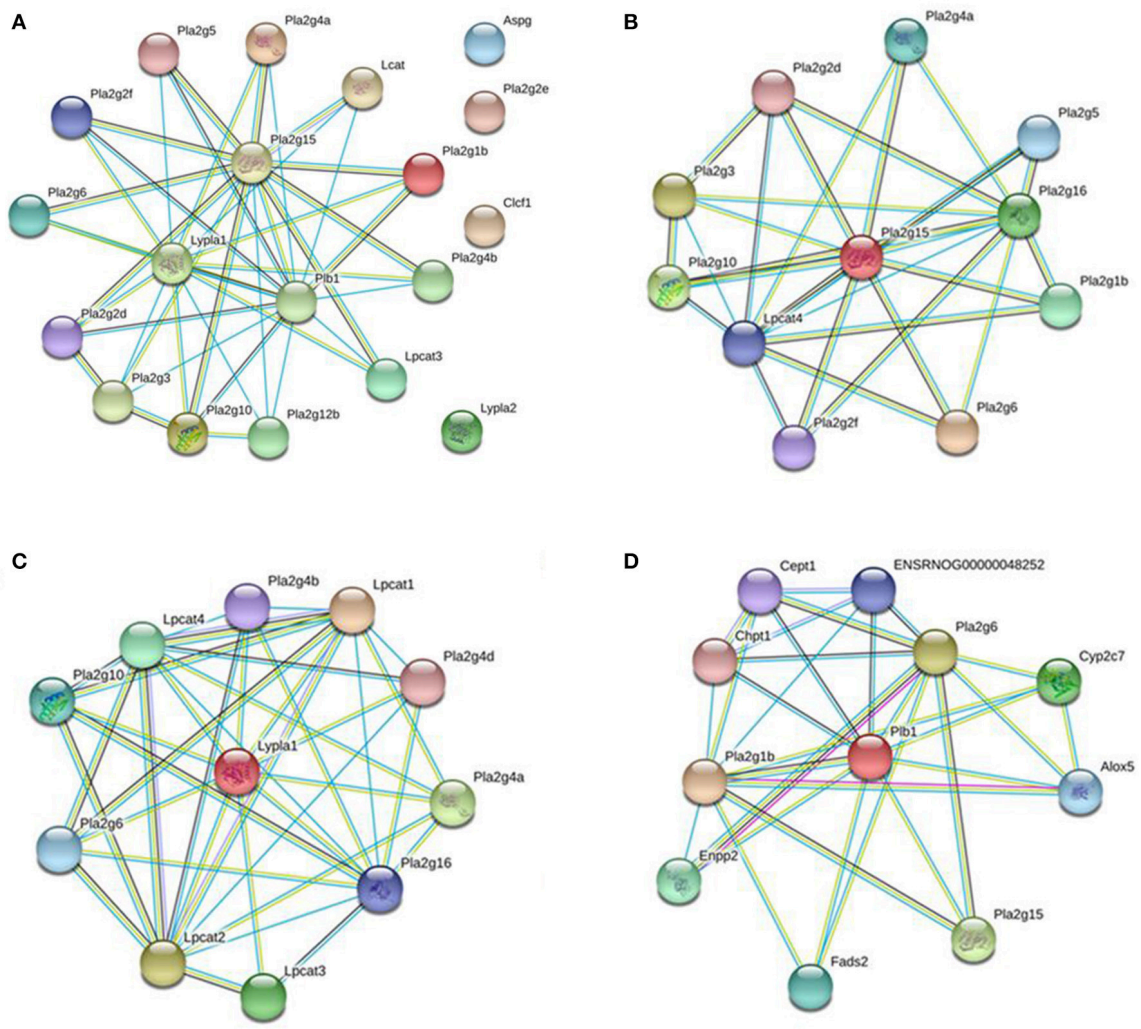
Interaction Networks of Enzymes and Proteins. **(A)** Functional interaction networks of the key enzymes were collected and input into the STRING database to analyze interactions between them (interaction score = 0.9). Pla2g15, lypla1, and plb1 were 3 most interactive enzymes. Functional interaction networks of pla2g15 **(B)**, lypla1 **(C)**, plb1 **(D)**. Only proteins with highest confidence (interaction score = 0.9) were considered as possible targets network for the metabolites.

**Figure 7:**
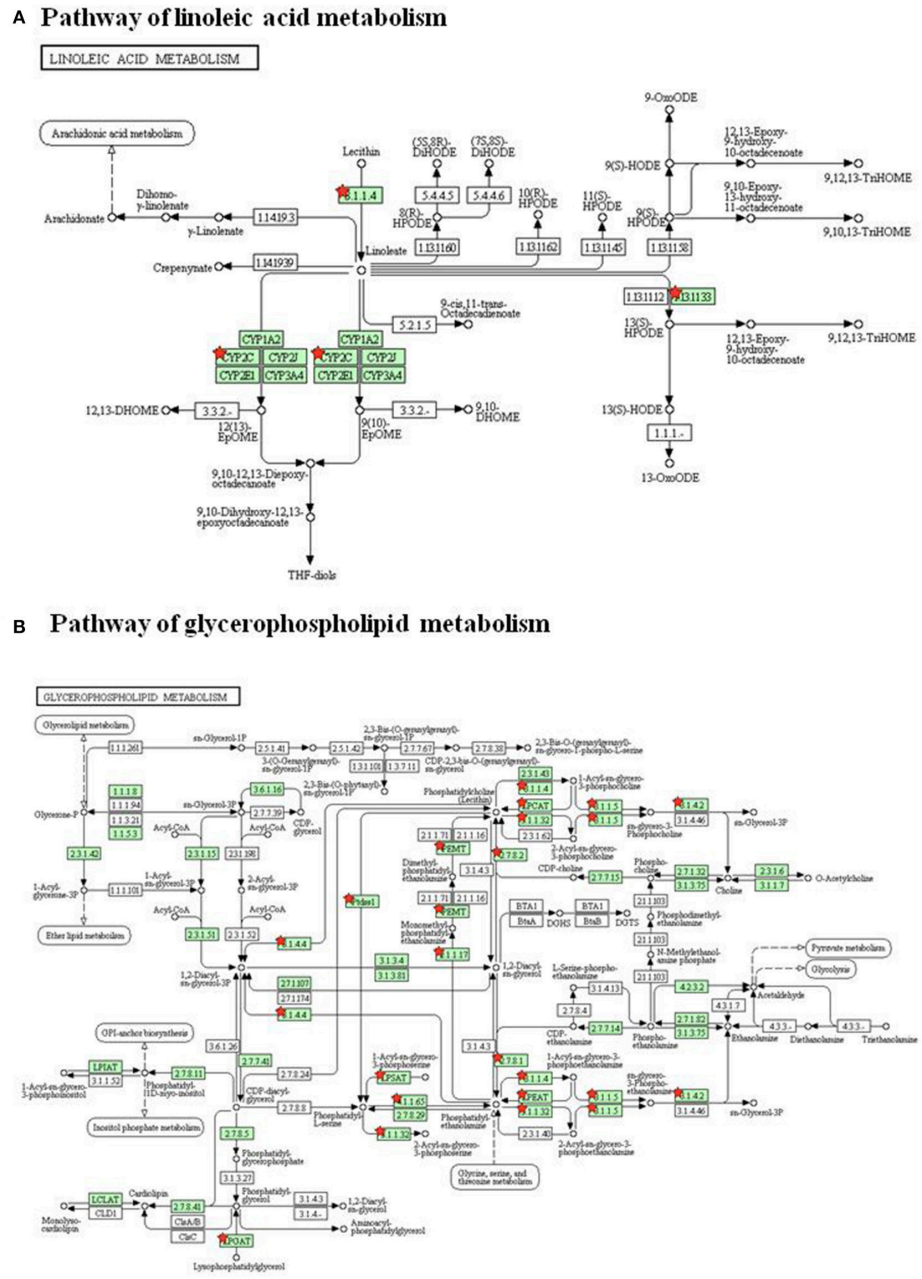
Biological Pathways of WLT treatment. **(A)** Pathway of linoleic acid metabolism. **(B)** Pathway of glycerophospholipid metabolism. The red stars mark potential targets of WLT in pathways.

To investigate the underlying mechanism of WLT, the metabolite–protein interaction networks were constructed. Nineteen enzymes related to the regulated metabolites were collected from HMDB (Table 3). Next, interaction networks of the enzymes and proteins were constructed by STRING. Interactions with highest confidence (interaction score = 0.9) were considered as active interactions (Figure 6A). As shown, pla2g15, lypla1, and plb1 were 3 most interactive enzymes. Their interaction networks of the enzymes and proteins were analyzed (Figures 6B–D). Correspondingly, the 27 proteins with highest confidence (interaction score = 0.9) were considered as possible targets network for the metabolites (Table 4). Additionally, the metabolite-related enzymes and proteins were mapped in the biological pathways to explore the metabolite-related signal pathways in DAVID 6.8. The 2 important biological pathways, including LA metabolism pathway, and glycerophospholipid metabolism pathway, were showed in Figure 7. The potential targets of WLT were marked in the pathways.

**Table 3:** Key enzymes of WLT treatment.

**Enzymes**	**Full name**	**Uniprot ID**
ASPG	60 kDa lysophospholipase	Q86U10
CLC	Eosinophil lysophospholipase	Q05315
LCAT	Phosphatidylcholine-sterol acyltransferase	P04180
LPCAT3	Lysophospholipid acyltransferase 5	Q6P1A2
LYPLA1	Acyl-protein thioesterase 1	O75608
LYPLA2	Acyl-protein thioesterase 2	O95372
PLA2G10	Group 10 secretory phospholipase A2	O15496
PLA2G12B	Group XIIB secretory phospholipase A2-like protein	Q9BX93
PLA2G15	Group XV phospholipase A2	Q8NCC3
PLA2G1B	Phospholipase A2	P04054
PLA2G2D	Group IID secretory phospholipase A2	Q9UNK4
PLA2G2E	Group IIE secretory phospholipase A2	Q9NZK7
PLA2G2F	Group IIF secretory phospholipase A2	Q9BZM2
PLA2G3	Group 3 secretory phospholipase A2	Q9NZ20
PLA2G4A	Cytosolic phospholipase A2	P47712
PLA2G5	Calcium-dependent phospholipase A2	P39877
PLA2G6	85/88 kDa calcium-independent phospholipase A2	O60733
PLB1	Phospholipase B1, membrane-associated	Q6P1J6
PLA2G4B	Cytosolic phospholipase A2 beta	P0C869

**Table 4:** Key proteins of WLT.

**Proteins**	**Full Name**	**Uniprot ID**
alox15	Arachidonate 12-lipoxygenase, leukocyte-type	Q02759
alox5	Arachidonate 5-lipoxygenase	P12527
cept1	Choline/ethanolaminephosphotransferase 1	Q6AXM5
chpt1	Cholinephosphotransferase 1	Q66H21
Cyp4f6	Cytochrome P450 4F6	P51871
cyp2c7	Cytochrome P450 2C7	P05179
lypla1	lysophospholipase 1	P70470
fads2	Fatty acid desaturase 2	Q9Z122
lpcat1	Lysophosphatidylcholine acyltransferase 1	Q1HAQ0
lpcat2	Lysophosphatidylcholine acyltransferase 2	P0C1Q3
lpcat3	Lysophospholipid acyltransferase 3	Q5FVN0
lpcat4	lysophosphatidylcholine acyltransferase 4	D3ZR52
pafah2	Platelet-activating factor acetylhydrolase 2, cytoplasmic	P83006
pla2g1b	Phospholipase A2	P04055
pla2g10	Group X secretory phospholipase A2	Q9QZT3
pla2g15	Group XV phospholipase A2	Q675A5
pla2g16	Group XVI phospholipase A1/A2	P53817
pla2g4a	Cytosolic phospholipase A2	P50393
pla2g4b	Cytosolic phospholipase A2 beta	D4A1I6
pla2g4d	cytosolic phospholipase A2 group IVD	D3ZQH6
pla2g5	Calcium-dependent phospholipase A2	P51433
pla2g6	85/88 kDa calcium-independent phospholipase A2	P97570
pla2g7	Platelet-activating factor acetylhydrolase	Q5M7T7
plb1	Phospholipase B1, membrane-associated	O54728
ptgs1	Prostaglandin G/H synthase 1	Q63921
ptgs2	Prostaglandin G/H synthase 2	P35355
ENSRNOG00000048252	Selenoprotein I	M0R5Z5

To further investigate the affinity between ingredients and potential targets of WLT, molecular docking was performed. A previous pharmaceutical study of WLT identified that HSYA, icariin, epimedin B and 4-DHBA (Table 5) penetrated through the skin, and they had significant protective effects on Schwann cells after injured by chemotherapy (Lin et al., 2017). Therefore, molecular docking was performed between these 4 active ingredients and the potential protein network to predict the probable targets of WLT against PIPN. The docking results revealed that there were high affinities between HSYA and alox15, alox5, cept1, cyp2c7, pla2g1b, pla2g10, pla2g4a, pla2g4d, pla2g7, and ptgs1. Similarly, high affinities were seen between Epimedin B, Icariin and alox5, chpt1, pla2g1b, pla2g4a, pla2g7, ptgs1, as well as between 4-DHBA and most key proteins except pafah2, pla2g16 (Table 6).

**Table 5:** Effective ingredients of WLT.

**Pub.Chem CID**	**Chemical Name**	**Molecular Formula**	**Molecular Weight (g/mol)**
6443665	Hydroxysafflor Yellow A	C_27_H_32_O_16_	612.537
5318997	Icariin	C_33_H_40_O_15_	676.668
5748393	Epimedin B	C_38_H_48_O_19_	808.783
1491	2,4-dihydroxybenzoic acid	C_7_H_6_O_4_	154.121

**Table 6:** Results of docking.

**Proteins**	**PDB ID**	**1491**	**5318997**	**5748393**	**6443665**
alox15	5ir4	16.1623	46.5708	NA	57.8408
alox5	3v99	15.7621	62.2073	71.1117	51.3596
cept1	5d92^*^	15.2359	NA	NA	34.4516
chpt1	5d92^*^	15.2041	37.4455	40.2172	NA
cyp4f6	5uec	21.0602	NA	NA	7.03
cyp2c7	5w0c	23.4282	NA	NA	64.0791
lypla1	5sym	26.2005	NA	NA	−9.59725
fads2	5xee^*^	13.479	NA	NA	NA
lpcat1	5b8i^*^	20.3833	NA	NA	−72.6425
lpcat2	5kym^*^	23.3109	NA	NA	NA
lpcat3	2lr8^*^	16.0385	NA	NA	NA
lpcat4	5kym^*^	16.7664	NA	NA	NA
pafah2	1vyh	−43.3331	NA	NA	NA
pla2g1b	1j1a	21.5994	63.113	63.7937	61.0024
pla2g10	5g3m	26.2186	NA	NA	49.1851
pla2g15	4x95	25.5394	NA	NA	NA
pla2g16	4fa0	−215.668	NA	NA	NA
pla2g4a	1cjy	27.2278	47.1398	57.0373	49.8278
pla2g4b	5iz5^*^	25.2277	NA	NA	NA
pla2g4d	5irz	16.0884	−40.2617	NA	58.2197
pla2g5	1fb2^*^	28.7858	NA	NA	NA
pla2g6	6aun	27.5994	NA	NA	NA
pla2g7	5lp1	20.3099	60.1397	55.1504	50.5743
plb1	5w7a^*^	25.9975	NA	NA	NA
ptgs1	1cqe^*^	14.1959	61.0679	67.2021	62.1509
ptgs2	5ikq	24.6126	NA	NA	NA

* *Template of homology modeling*.

## Discussion

CIPN is a serious dose-limiting toxicity of many anti-neoplastic agents, especially paclitaxel and oxaliplatin. Because the mechanisms are not explicit, there is hardly any effective agent for the prevention or management of CIPN. Previous studies showed that WLT, a topical applied ointment with herbal formula, was helpful to prevent and alleviate CIPN. This study was aimed to explore the mechanisms behind it. We identified 19 significant changed metabolites of PIPN, most of which were lipids (LysoPCs or PCs), organic acids, and ketones. Metabolism pathway study demonstrated that paclitaxel induced peripheral neuropathy mainly by targeting LA metabolism pathway and glycerophospholipid metabolism pathway. Correspondingly, WLT attenuated symptoms of PIPN by primarily rebalancing these two pathways. As a pilot study, we explored the methodology of metabolomics and network pharmacology on PIPN. The findings of pathways and potential targets might provide important clues for further mechanism studies and clinical improvement.

CIPN is a complex topic. Although the anti-neoplastic features are well described, no explicit pathophysiological process can be identified to explain the various neuropathies (Addington and Freimer, 2016). The neurotoxic side effects may be related to multifactorial metabolism pathways. Studies have documented that processes of neurotransmitter signaling, such as glutamate and γ-aminobutyric acid, are involved with CIPN occurrence (Cata et al., 2006; Carozzi et al., 2015). Caspase signaling is also found to contribute to PIPN, leading to the generation of reactive oxygen species (ROS), as well as potential apoptosis (Park et al., 2004). Additionally, pathways activate inflammatory cytokines, such as TNF-α, MAPK, and CX3CL1, are involved with CIPN as well (Cavaletti et al., 2000; Zhang et al., 2010; Brandolini et al., 2017). Moreover, recent evidences indicated that activation of TRPV1 during chemotherapy sensitizes pain pathways. Recent studies identified members of the TRP family of ion channels (TRPV1, TRPA1, and TRPV4) as contributors to mechanical allodynia during PIPN. However, it remains unclear which endogenous mediators were involved with paclitaxel-induced activation or sensitization of TRP channels, as paclitaxel cannot directly activate TRP channels (Alessandri-Haber et al., 2008; Chen et al., 2011; Jang et al., 2012; Materazzi et al., 2012; Hara et al., 2013; Boyette-Davis et al., 2015; Li Y. et al., 2015). In present study, we found that LA metabolism pathway was a key pathways contributed to PIPN. LA is a doubly unsaturated fatty acid, also known as an omega-6 fatty acid. It is used in the biosynthesis of prostaglandins (via arachidonic acid) and cell membranes. This finding is supported by other studies. Paclitaxel is an inducer of some Cytochrome-P450 epoxygenases (e.g., CYP2C8, CYP2C9), which can metabolize ω-6 fatty acids, such as arachidonic acid (AA) and LA (Dai et al., 2001). Another study identified that 9,10-EpOME (9,10-epoxy-12Z-octadecenoic acid), a CYP metabolite of LA, was strongly synthesized in DRGs after paclitaxel injection. Injection of 9,10-EpOME alone caused significant reduction of the mechanical thresholds of wild-type mice too. Further, decreasing 9,10-EpOME concentrations in DRGs and in plasma reversed mechanical hypersensitivity in paclitaxel treated mice (Sisignano et al., 2016). Glycerophospholipid are the main component of neural membranes. The main function of glycerophospholipids in the neural membranes is to provide stability, permeability and fluidity through specific alterations in their compositions (Glynn, 2013; Ruiz et al., 2015). Previous study demonstrated that the proportion of glycerophospholipid decreased significantly in diabetic neuropathy rats (Kuruvilla and Eichberg, 1998).

According to TCM theory, CIPN was blamed to *Meridian obstruction due to cold congealing*. Consequently, based on syndrome differentiation and the holistic theory, WLT was developed to act the effect of *Warming and Activating Meridian, Promoting Blood Circulation, and Alleviating Pain*. It was constitute of four herbs, which were epimedium herb, geranium wilfordii, cassia twig, and carthamus tinctorius. Respectively, epimedium herb, acting as *Monarch* (the most important constituent), was used to *tonify Kidney Yang* and *dispel Wind-Damp*. Geranium wilfordii, acting as *Minister* (the second important constituent), was used to *expel Wind* and *dredging Collat*. cassia twig, acting as *Assistance* (a constituent helps *Monarch* or/and *Minister* to achieve their effects), was used to w*arm and activate Meridian* and *reinforce Yang to promote the flow of Qi*. Carthamus tinctorius, acting as *Assistance* (a constituent helps *Monarch* or/and *Minister* to achieve their effects), was used to *promote blood circulation and remove Meridian obstruction*. The herbs of WLT were supposed to act synergistically and achieve the effect of *Warming and Activating Meridian, Promoting Blood Circulation and Alleviating Pain*. Meanwhile, some pharmacological studies of herbs also indicated that constituents of WLT could be helpful for alleviating neuropathy (Kou et al., 2013; Sun et al., 2016). A study showed that epimedium extract promotes peripheral nerve regeneration in rats (Oh et al., 2015). Another study showed icariin, a flavonoid glycosides extracted from epimedium herb, promoted the expression of nNOs after neural injury (Shindel et al., 2010). It was also reported that cassia twig, geranium wilfordii and carthamus tinctorius had anti-inflammatory, antioxidative, neurorotective and analgesic effects (Gan et al., 2010; Wang et al., 2014; Yue et al., 2014; Gunawardena et al., 2015; Huang et al., 2015; Jo et al., 2017). According to previous study, we took 4 active ingredients of WLT as probe to conduct molecular docking with potential targets obtained from metabolomic analysis. Published literatures showed their neuroprotective effects. A study revealed that HSYA showed neuroprotection through attenuating oxidative stress, inflammatory response, and neural apoptosis (Pei et al., 2017). Another study showed that Icariin could be a potential agent for the treatment of paclitaxel-induced neuropathic pain in a SIRT1-dependent manner (Gui et al., 2018). Another study suggested that 3,4-DHBA prevented neuronal cell damage by interfering with the increase of Ca^2+^ (Ban et al., 2007). The docking results showed complex interactions between them, demonstrating the characteristics of multi-components and multi-targets. The ingredients showed high affinity to most proteins, except pafah2, pla2g16, and ENSRNOG00000048252. Some proteins showed complex interactions with more than 1 ingredient, such as alox15, alox5, cept1, chpt1, cyp4f6, cyp2c7, pla2g1b, pla2g10, pla2g4a, pla2g4d, pla2g7, and ptgs1. The acting sites in LA pathway and glycerophospholipid pathway of these proteins were marked in Figure 7.

## Conclusion

In this study, LC-MS based non-targeted metabolomics method revealed the metabolic disturbances induced by paclitaxel, and the effects of WLT on alleviating PIPN. Paclitaxel primarily disturbed the LA and glycerophospholipid metabolism pathways to induce peripheral neuropathy. Further study demonstrated that WLT attenuated PIPN by regulating these two pathways in the manner of multi-target interference.

## Author contributions

FW participated in research design, carried out most of the studies, performed statistical analysis, and wrote the manuscript. WX performed data analysis and contributed to the writing and modification of the manuscript. BD and CD provided professional advices for research design and manuscript revising. SL, MW, and YG participated in the animal experiments. LJ provided professional advices for research design, statistical analysis, and manuscript revising.

### Conflict of interest statement

The authors declare that the research was conducted in the absence of any commercial or financial relationships that could be construed as a potential conflict of interest. The reviewer JC declared a shared affiliation, with no collaboration, with one of the authors, FW, to the handling editor at the time of the review.
