# Supplementary material for: Wen-Luo-Tong Decoction Attenuates Paclitaxel-Induced Peripheral Neuropathy by Regulating Linoleic Acid and Glycerophospholipid Metabolism Pathways
Source: Front Pharmacol. 2018 Aug 28;9:956. doi: 10.3389/fphar.2018.00956 (PMC6127630; doi:10.3389/fphar.2018.00956)
Supplement: Supplementary file 1 [file Image_1.pdf]

Supplementary Figure 1: Process of WLT preparation

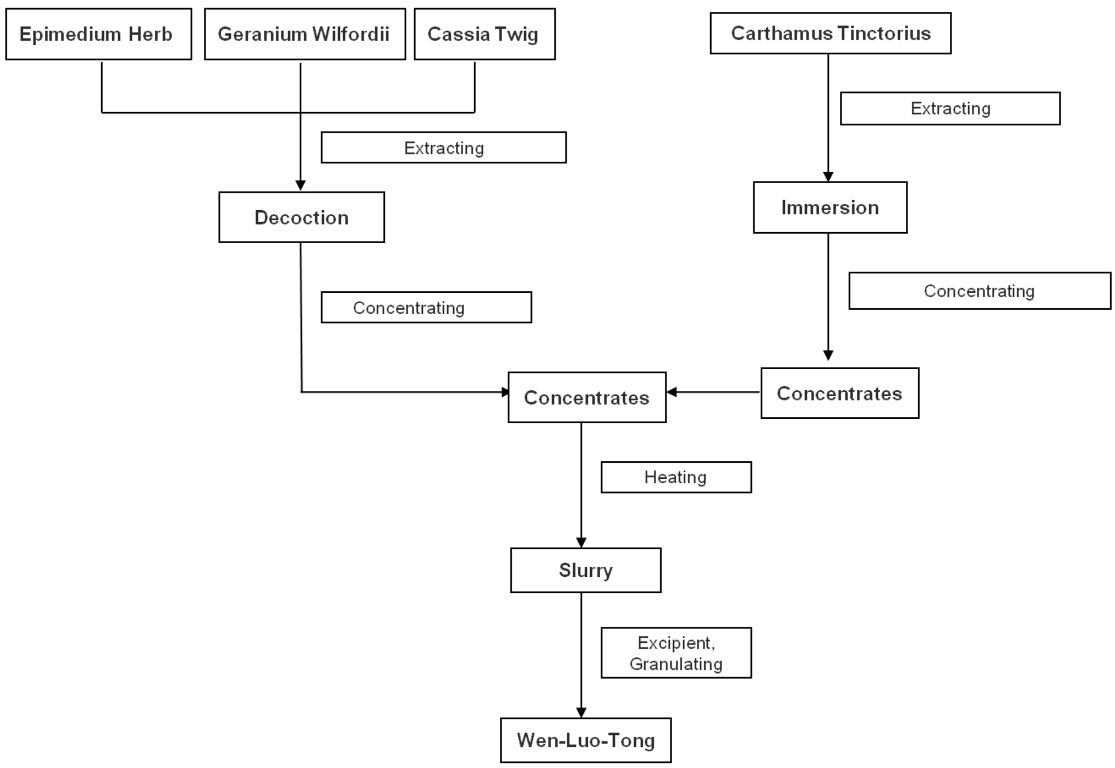

Supplementary Figure 2: Principal component analysis of samples.

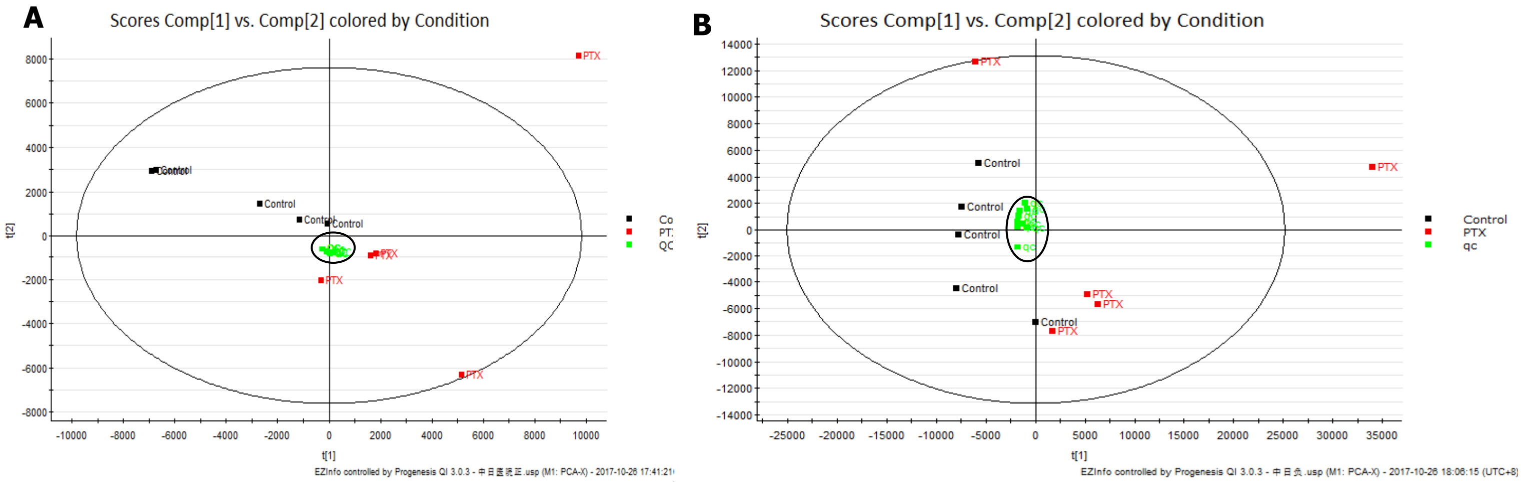

(A) PCA plot of negative mode

$R^2X = 0.983$, $Q^2=0.820$

(B) PCA plot of negative mode

$R^2X = 0.795$, $Q^2=0.473$

To monitor system's stability and performance of QC samples, they were analyzed along with other samples by PCA**.** The PCA plots showed a clear discrimination between Control and PTX groups, while QC samples was highly clustered, indicating the paclitaxel induced peripheral neuropathy model was successfully duplicated and our analytical method was of good reproducibility and stability.

Supplementary Figure 3:

(A) Key metabolites of linoleic acid metabolism

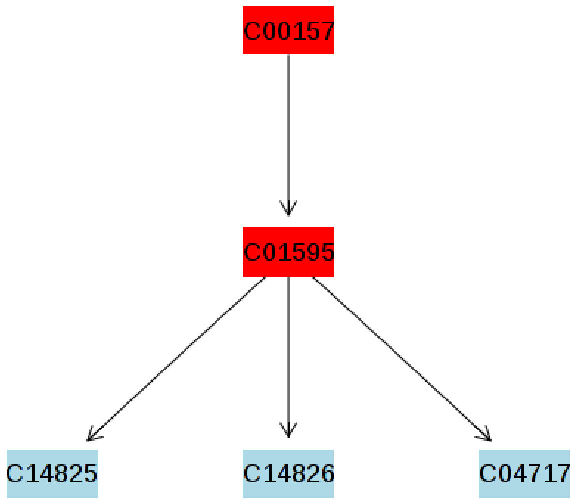

(B) Key metabolites of glycerophospholipid metabolism

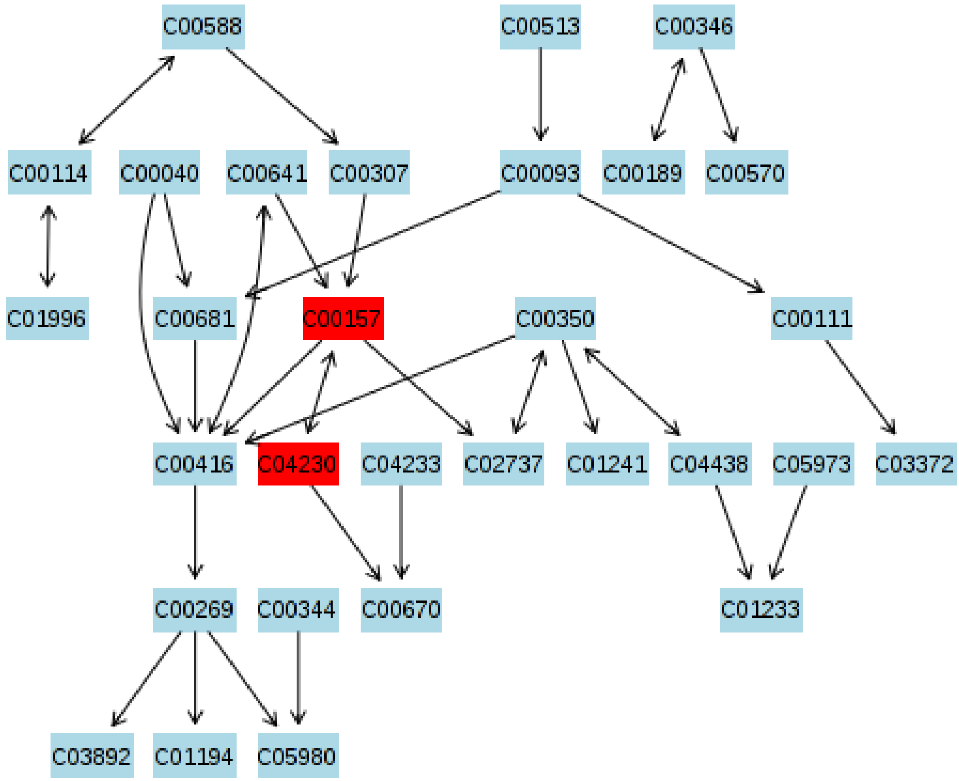

Supplementary Figure 4:

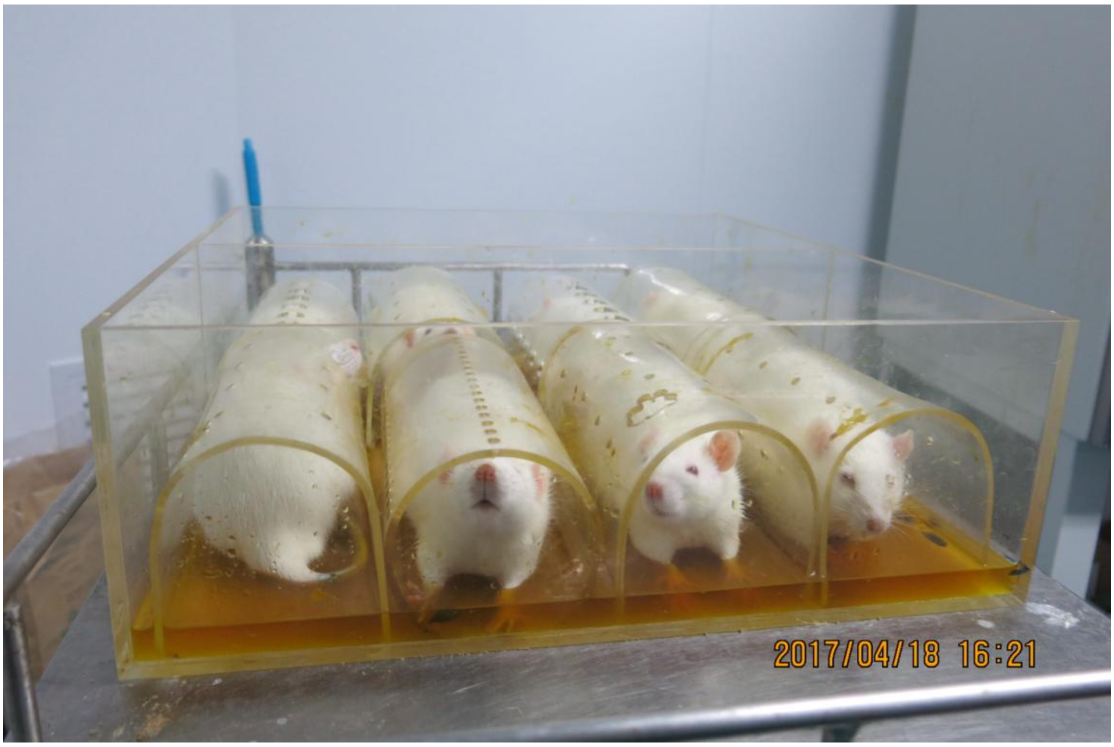

Rats were confined in special chambers separately while taking WLT/water pediluvium. Rats in the same group shared one batch of WLT solution/water every time.
